# P-781. Evaluating UTI Management Using a Proposed Continuum of Diagnosis in an Academic Medical Center

**DOI:** 10.1093/ofid/ofaf695.992

**Published:** 2026-01-11

**Authors:** Parker Kaleo, Alaina DeKerlegand, Dennis Marjoncu, Carolyn Cummings, Kerry O Cleveland

**Affiliations:** Tampa General Hospital, Tampa, FL; Methodist University Hospital, Memphis, Tennessee; Methodist University Hospital, Memphis, Tennessee; Methodist University Hospital, Memphis, Tennessee; University of Tennessee Health Science Center, Memphis, Tennessee

## Abstract

**Background:**

Controversy remains when managing urinary tract infections (UTIs) for patients without urinary symptoms but with nonspecific clinical symptoms. A new diagnostic category was recently proposed termed bacteriuria of unclear significance (BUS) to acknowledge this gap and shine light on treatment decisions in these patients. We evaluated this classification in our academic medical center by retrospectively reviewing patients who were diagnosed and treated with a UTI as having asymptomatic bacteriuria (ASB), BUS, or a UTI.

**Figure 1.**
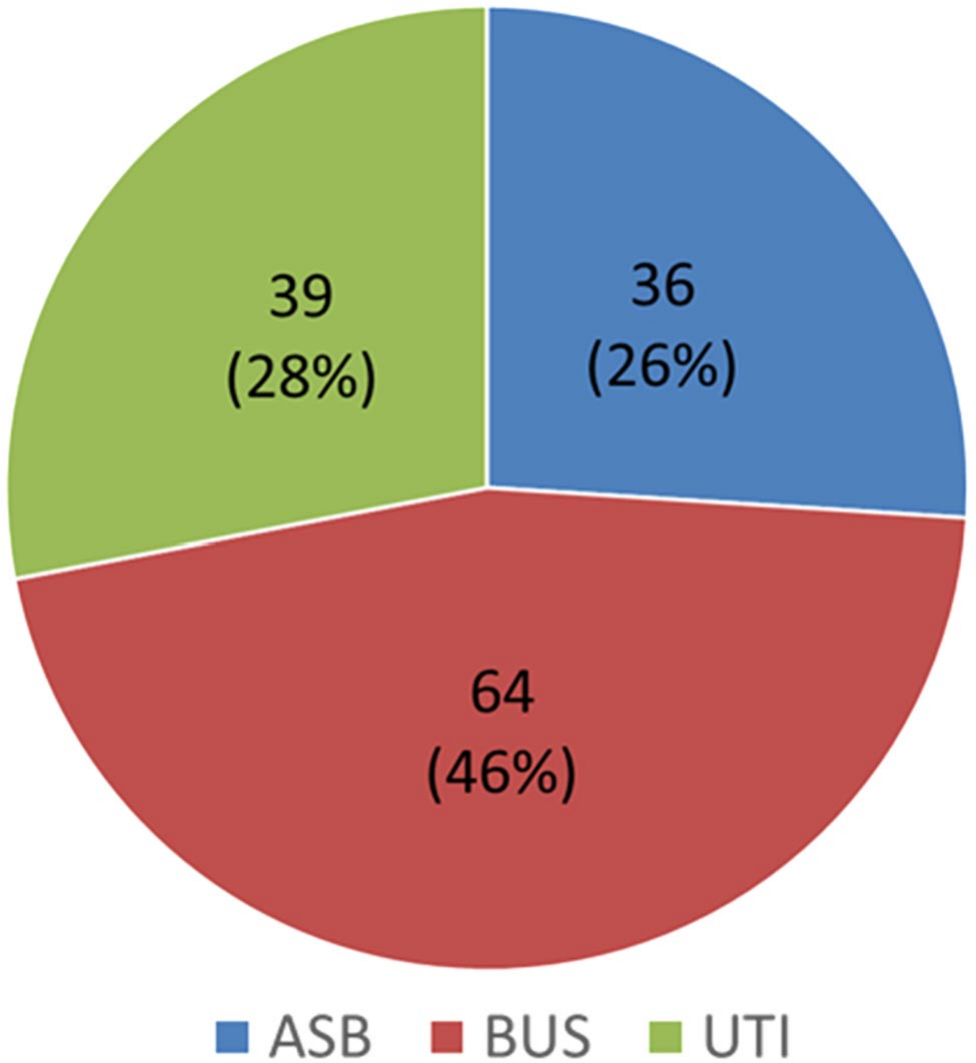
Primary Objective

**Table 1.**
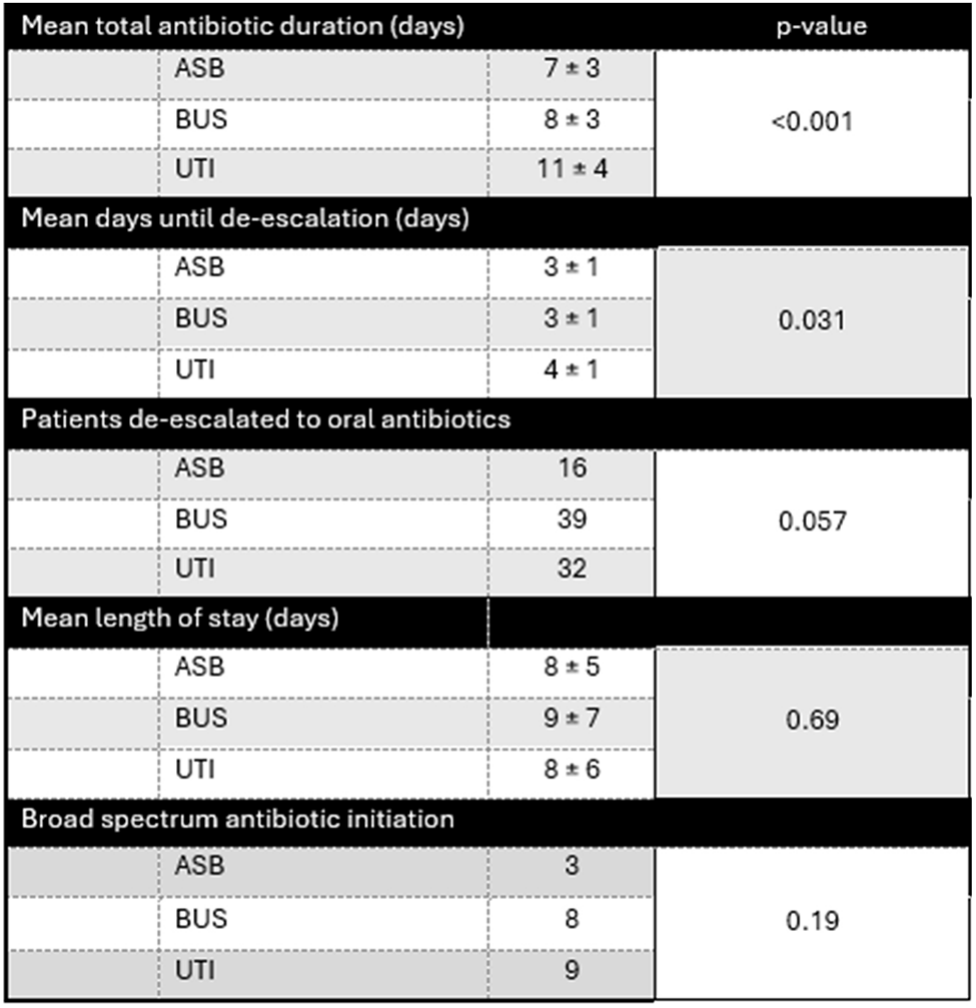
Primary and Secondary Outcomes

**Methods:**

This retrospective study included adults (≥ 18 years) diagnosed with a UTI from July 1, 2023, to July 31, 2024. Patients had positive urinalysis, positive urine cultures, and received antibiotics for > 24 hours. Patients with complicating factors were excluded.

The primary objective was to re-categorize patients into ASB, BUS, or UTI and compare antibiotic duration between categories. Secondary outcomes included de-escalation rates, time to de-escalation, length of stay, and broad-spectrum antibiotic use.

**Results:**

A total of 139 patients were included with a median age of 70 years and 59% being female. Only 23 (17%) had urinary symptoms and the most common antibiotics were ceftriaxone empirically (81%) and levofloxacin for oral de-escalation (33%). Overall, 26% were re-categorized with ASB, 46% with BUS, and 28% with UTIs (Figure 1).

The mean antibiotic duration was 7 days for ASB, 8 days for BUS, and 11 days for UTIs (p < 0.001)(Table 1). The mean time to de-escalation was 3 days for ASB and BUS, and 4 days for UTI (p=0.031). De-escalation rates were 44%, 61%, and 82% (p=0.057), respectively for ASB, BUS, and UTI. Length of stay averaged 8 days for ASB, 9 for BUS, and 8 for UTI (p=0.69). Patients initiated on broad spectrum antibiotics were 3 in the ASB group, 8 in BUS, and 9 in UTI (p=0.19).

**Conclusion:**

Nearly half of patients were re-categorized as having BUS and were treated more similarly overall to those with ASB than with UTIs. Further research on clinical and microbiological outcomes are required to evaluate appropriate treatment methods for this novel diagnostic category. This study emphasizes the need for education and antimicrobial stewardship protocols to address antibiotic overuse in ASB and overtreatment of UTIs.

**Disclosures:**

Kerry O. Cleveland, MD, AbbVie Pharmaceuticals: Honoraria|Merck Pharmaceuticals: Honoraria

